# Enabling generalizable RUL prediction for equipment: a dual-dimensional continual learning approach based on deep Gaussian process

**DOI:** 10.1038/s41598-026-62444-z

**Published:** 2026-08-07

**Authors:** Liu Xiuli, Chen Zifu, Wu Guoxin, Cui Shuo

**Affiliations:** 1 Key Laboratory of Modern Measurement & Control Technology, Ministry of Education, Beijing Information Science and Technology University, Beijing, 100192 China; 2 School of Mechanical and Electrical Engineering, Beijing Information Science and Technology University, No. 12, Xiaoqingying East Road, Haidian District, Beijing, 100192 China

**Keywords:** Continual learning, Cross-operating conditions, Uncertainty quantification, Remaining useful life prediction, Deep learning, Engineering, Mathematics and computing

## Abstract

Accurately predicting Remaining Useful Life (RUL) is critical for the intelligent maintenance of high-end rotating machinery, which often operates under complex and variable conditions. However, conventional predictive models tend to degrade in performance when deployed in novel scenarios due to domain shifts. This study proposes a novel predictive framework that integrates Deep Gaussian Processes (DGP) with a dual-dimensional constrained continual learning (CL) strategy to address these challenges. Specifically, the framework leverages the probabilistic nature of DGP to quantify the inherent uncertainty during degradation. To preserve cross-domain degradation knowledge, we employ Elastic Weight Consolidation, which anchors critical weights, and Gradient Episodic Memory, which corrects update directions in the gradient space that conflict with established degradation patterns. Together, these techniques form a dual-dimensional constrained CL framework. This synergistic approach enables the DGP to incrementally assimilate knowledge from different operating conditions (cross-condition) and diverse machine types (cross-device) without suffering from catastrophic forgetting. Experimental results demonstrate that the proposed framework not only exhibits outstanding resistance to catastrophic forgetting and excellent multi-scenario adaptability, but also achieves superior predictive accuracy compared to state-of-the-art methods by leveraging the dual-dimensional continual learning strategy. Furthermore, the integrated DGP module provides robust uncertainty quantification, offering a reliable basis for intelligent maintenance decision-making in complex operating environments.

## Introduction

Driven by the rapid evolution of Industry 4.0 and smart manufacturing, Prognostics and Health Management (PHM) has become a critical strategic pillar for industrial equipment, particularly in the prediction of Remaining Useful Life (RUL)^1^. RUL prediction plays a crucial role in assessing equipment health, monitoring degradation trajectories, and optimizing maintenance strategies^2^. Accurate RUL estimations enable a shift to proactive maintenance, minimizing downtime-related economic losses, optimizing resource allocation, and improving overall operational profitability.

However, in practical industrial settings, mechanical equipment often faces complex operating conditions, multiple load couplings, and external disturbances^3^. Its degradation process is characterized by strong nonlinearity, non-stationarity, and random fluctuations^4^. As industrial equipment scales up and the diversity of equipment types increases, cross-device RUL prediction has become a key research focus. Due to significant variations in structural parameters, operational environments, sensor configurations, and signal patterns across different devices, single-device models are often difficult to transfer directly to other equipment^5^. To address the problem of poor model generalization across diverse working conditions, optimized and multistage transfer learning strategies have been widely adopted for rotating machinery fault diagnosis and health monitoring^6,7^. Nevertheless, existing transfer learning methods still struggle to maintain stable performance in complex cross-device scenarios. As a result, achieving reliable and robust RUL prediction under cross-operating-condition and cross-device scenarios has become a major scientific challenge and technical bottleneck in contemporary intelligent maintenance systems^8^.

In recent years, deep learning methods have shown considerable promise in RUL prediction tasks. Convolutional Neural Networks (CNN)^9^ can automatically extract local degradation features, while Recurrent Neural Networks (RNNs)^10^ and Long Short-Term Memory (LSTM) networks^11^ are well-suited for modeling long-term dependencies in time series. Time-Convolutional Networks (TCN)^12^ and Transformers^13^ excel at efficiently capturing multi-scale degradation dynamics. Furthermore, TCN-based systems have also been verified effective for early fault detection and smart predictive maintenance of industrial machinery^14^. These approaches significantly enhance the representational capacity and predictive performance of degradation features, providing a solid foundation for RUL prediction under complex operating conditions. However, deep learning methods typically rely on offline training with fixed datasets. In real industrial settings, equipment data is acquired incrementally, with variable operating conditions and changing life stages. Therefore, models must continuously learn from new tasks without complete retraining, making CL a critical research direction in RUL prediction^15,16^.

CL represents an effective learning paradigm enabling deep networks to retain prior knowledge whilst acquiring new insights, thereby mitigating catastrophic forgetting. In recent years, with the phased accumulation of mechanical failure data within the industrial Internet of Things, CL has garnered increasing attention in mechanical RUL prediction, offering novel pathways for predictive tasks in dynamic data scenarios. Xu et al. introduced an experience-dependent adaptive synaptic scaling mechanism for spiking neural networks, utilizing gradient signal regulation to facilitate long-term memory consolidation and significantly enhance robustness in continual learning^17^. Kong et al. introduced EWC into RUL prediction, employing a regularization term to penalize significant weight changes^18^; Wang et al. combined EWC with reserve computation to further validate the efficacy of parameter constraints in preserving memory^19^ . Zeng et al. proposed an orthogonal weight correction method, restricting weight update directions via gradient projection to minimize disruption to prior knowledge^20^. Qin et al. constructed a CL framework based on generalized learning, adapting networks to new data distributions through incremental node-adaptive updates^21^.

Concurrently, probabilistic modeling—as a key technique for quantifying prediction uncertainty—has demonstrated significant advantages in mechanical RUL forecasting. Given the inevitable noise interference in sensor data and the dynamic evolution of degradation patterns, uncertainty quantification emerges as a core requirement for enhancing prediction reliability. To improve the reliability of industrial fault detection and prediction, physics-guided learning and model calibration strategies have also been combined with deep networks to achieve more trustworthy early warning results^22^. Existing research primarily employs two categories of probabilistic modeling approaches. The first category is Bayesian modeling approaches, where Lin et al.^23^ proposed a generalized RUL prediction method based on mixture models, innovatively constructing a sparsity mechanism via sparse variational Bayesian methods. Yasong Li et al. (2025) developed a self-data-driven framework combining "Bayesian Attention + State Transition", with the core innovation being the Bayesian Attention Module for RUL prediction^24^. Another category comprises probabilistic generative models: Chen et al. (2023) integrated the nonlinear modeling capability of DGP with the state recognition capability of Hidden Markov Models^25^ . Zhan et al.^26^ proposed a "Multi-Distribution Fusion" architecture, simultaneously utilizing multiple prediction distributions to characterize RUL uncertainty.

Although the aforementioned methods achieve the basic goals of "learning new without forgetting old" and uncertainty quantification in specific scenarios, they face higher demands for robustness in CL and reliability in uncertainty quantification when applied to RUL prediction under complex mechanical operating conditions and cross-device scenarios. Existing RUL prediction methods for mechanical systems still have the following limitations:

1. In real-world industrial scenarios, degradation patterns are influenced not only by the natural aging process but also by sudden changes in operating conditions and inherent differences between different power generation units. Methods that rely solely on parameter importance struggle to consolidate knowledge under these complex domain shifts, as the “importance” of parameters becomes unstable in non-stationary tasks. Wang et al. point out that the parameter ‘importance’ evaluated in early tasks not only becomes invalid under drastic distribution shifts, but this rigid constraint also completely stifles the plasticity required for the model to adapt to new scenarios^27^. On the other hand, methods that rely solely on episodic memory may find that their limited memory buffer is insufficient to represent the significant variations in degradation signals across multiple operating domains. Aggarwal et al. note that limited by strict memory budgets, experience replay algorithms cannot accommodate a sufficiently diverse set of historical samples within their restricted buffer capacities^28^. As a result, these single-constraint approaches often lead to poor knowledge transfer, thus limiting their application in real-world deployments.
2. Existing cross-domain methods primarily focus on feature alignment or domain adaptation, aiming to enhance the transfer effect for improved predictive accuracy. However, they generally overlook the evaluation of the reliability of the predictions. As Abdar et al. point out, cross-domain prediction inherently faces dual uncertainty: distribution differences between domains increase cognitive (epistemic) uncertainty for novel conditions, while sensor noise and operational fluctuations amplify incidental (aleatoric) uncertainties^29^. Furthermore, Peng et al. highlight that most current deep learning research on RUL prediction adopts deterministic neural networks, which can only output point estimates and fail to provide confidence intervals or probability distributions for predictions^30^.

To overcome the limitations mentioned above, this paper proposes a dual-dimensional constrained continual learning framework for RUL uncertainty quantification, aimed at mechanical RUL prediction under complex operating conditions and cross-device scenarios. The main contributions are as follows:

1. Hybrid constraint continual learning mechanism for complex operating conditions: This paper proposes a dual-dimensional continual learning strategy that combines EWC and GEM^31^. This approach overcomes the limitations of traditional single-dimensional constraints by applying regularization to key model parameters via EWC, preserving the degradation knowledge from previously learned tasks. Meanwhile, GEM is introduced to constrain the gradient directions, ensuring that the model can successfully transfer knowledge when learning new operating conditions or new equipment without forgetting old knowledge. This dual-constraint mechanism enables the model to maintain stable learning ability and predictive performance, even when facing frequent load fluctuations, environmental changes, and differences in equipment types in complex operational environments.
2. Cross-domain RUL prediction framework with uncertainty quantification: In response to the general neglect of prediction reliability evaluation in current cross-domain RUL prediction research, this paper establishes an uncertainty quantification framework based on DGP, incorporating probabilistic modeling into the continual learning tasks of cross-domain prediction for the first time. This method utilizes the powerful nonlinear fitting ability of DGP to extract degradation features, while its probabilistic output characteristics explicitly model the inherent uncertainties in cross-domain prediction.

The remainder of this paper is structured as follows. Section “Preliminary” introduces preparatory knowledge. Section “A Deep Gaussian process-based TWO-dimensional constrained continual learning framework for mechanical RUL prediction” details the proposed CL framework. Section “Experimental verification” validates the framework through bearing and Wind Turbine Gearbox remaining useful life predictions alongside ablation experiments, comparing results with existing state-of-the-art methods. Finally, Section “Conclusion” summarizes the contributions.

## Preliminary

### Continual learning

Deep neural networks frequently encounter catastrophic forgetting when sequentially learning new tasks, making continual learning a crucial research direction for addressing this issue^27^. Existing approaches primarily fall into three categories: regularization-based methods suppress excessive updating of critical weights through parameter importance constraints^18^ ; parameter isolation approaches preserve knowledge by freezing parameters from old tasks or extending new network branches^32^ ; while replay-based methods recreate past task experience through sample storage or generation^33^ .

### Gaussian process

Gaussian Processes (GP)^34^ differ from traditional parametric models in that they do not directly learn fixed-dimensional parameters. Instead, they model the distribution of a function, enabling high-confidence predictions and uncertainty estimation even with limited data. In Gaussian Processes, given an input *X* and its corresponding output *y* , the function *f(x)* follows the prior distribution:

1.1 *f(X) ∼ GP(m(X),k(X,X))*

where m(x) is the mean function (typically set to 0), and $k(\cdot ,\cdot )$ is the covariance function. Given a new input ${X}_{*}$ , the joint Gaussian distribution of the GP can be expressed as:

1.2 $$\left[ {\begin{array}{*{20}c} y \\ {[4pt]f_{*} } \\ \end{array} } \right]\sim {\mathcal{N}}\left( {0,\;\left[ {\begin{array}{*{20}c} {K(X,X) + \sigma^{2} I} & {K(X,X_{*} )} \\ {[6pt]K(X_{*} ,X)} & {K(X_{*} ,X_{*} )} \\ \end{array} } \right]} \right)$$

Through Bayesian inference, the posterior predicted mean and uncertainty for test point ${X}_{*}$ can be obtained: Predicted mean:

1.3 $$\mu_{*} = K(X_{*} ,X){\mkern 1mu} (K(X,X) + \sigma^{2} I)^{ - 1} y$$

Predicted variance:

1.4 $$\begin{gathered} \sigma_{*}^{2} = K(X_{*} ,X_{*} )\; - \;K(X_{*} ,X){\mkern 1mu} (K(X,X) + \sigma^{2} I)^{ - 1} \\ *{\mkern 1mu} K(X,X_{*} ) \\ \end{gathered}$$

GP provides point predictions alongside variance-based uncertainty estimates during forecasting, conferring advantages in industrial health monitoring, reliability assessment, and small-sample learning environments. However, its computational complexity grows exponentially with sample size ($O({N}^{3})$ ), rendering native GP impractical for large-scale applications.

## A deep Gaussian process-based two-dimensional constrained continual learning framework for mechanical RUL prediction

### Deep Gaussian processes-based two-dimensional constrained CL framework

**Fig.1:**
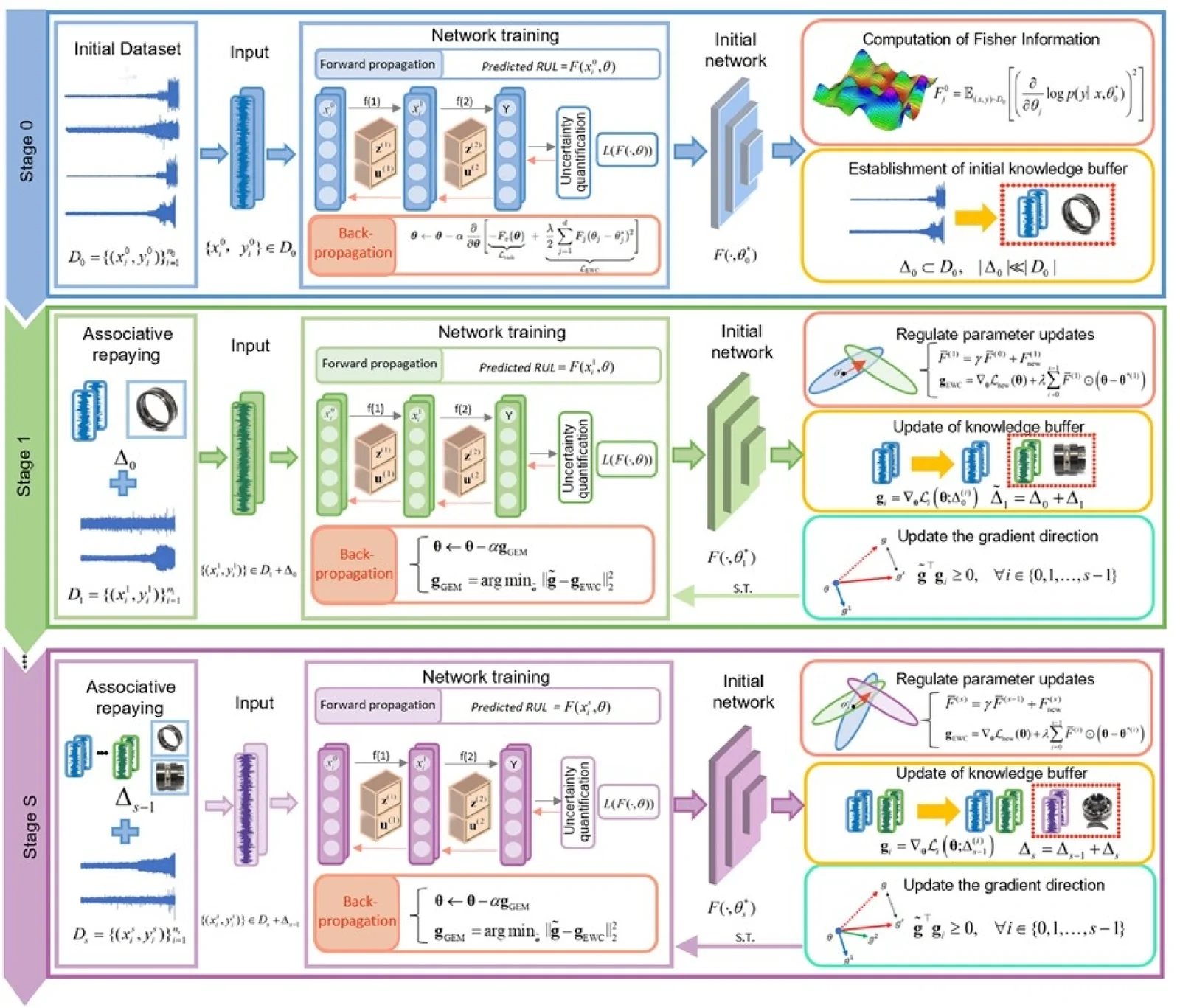
A dual-dimensional CL approach based on deep Gaussian process.

This paper constructs a dual-constraint CL framework for mechanical RUL prediction. The proposed framework features two characteristics: uncertainty quantification and dual-constraint. Uncertainty-quantified prediction outputs not only achieve higher accuracy but also comprehensively quantify overall uncertainty. The dual-constraint mechanism ensures core degradation patterns remain intact while preventing new task learning from compromising prior knowledge. The overall workflow of the proposed framework, as illustrated in Fig. 1, is systematically organized into the initial phase (Stage 0) and the subsequent continual learning phases (Stage S). The mathematical description of these functional blocks is summarized as follows:

#### Initial phase(Stage 0):

The proposed continual learning framework performs RUL on a sequence of tasks stream ${\mathcal{T}} = \{ T_{1} ,T_{2} , \ldots ,T_{S} \}$. For each current task ${T}_{s}$, the objective is to learn from the new task dataset ${D}_{s}$ while optimizing the parameters *θ* of the DGP, so as to preserve the knowledge acquired from previous tasks $\{ T_{1} , \ldots ,T_{s - 1} \}$ to the greatest extent possible.

In the initial phase, define the degradation dataset ${D}_{0}={\{({x}_{i}^{0},{y}_{i}^{0})\}}_{i=1}^{{n}_{0}}$.where ${n}_{0}=\left|{D}_{0}\right|$,${x}_{i}^{0}\in R$ denotes the feature vector of the *i*-th sample, and ${y}_{i}^{0}\in R$ represents the corresponding RUL label. Based on a DGP, where the DGP network is denoted as $F\left(\bullet ,\theta \right)$, it undergoes initial training to obtain optimized parameters:${\theta}_{A}^{*}$.The aforementioned DGP uncertainty quantification process will be detailed in Section "Uncertainty Quantification with Dual-Dimensional Spatial Constraints". Following initial training, the following two operations are performed:

(1) Fisher information computation:

The importance of the initial parameters is computed and represented in the form of a Fisher information matrix:

2.1 $$F_{j}^{0} = {\mathbb{E}}_{{(x,y)\sim D_{0} }} \left[ {\left( {\frac{\partial }{{\partial \theta_{j} }}\log p(y\left| {x,\theta_{0}^{*} } \right.)} \right)^{2} } \right],\quad j = 1, \ldots ,Z$$

where *F*0 jdenote the *j*-th element of the Fisher information matrix under task 0; Z represents the number of trainable parameters (synaptic weights) in the network. Fisher information reflects the contribution of parameter c to the initial task and serves as a stabilizing constraint in subsequent stages.

(2) Establishing the Knowledge Buffer:A small subset of representative and diverse samples is selected from the initial dataset ${D}_{0}$ to construct the initial knowledge buffer: ${\Delta}_{0}\left|{\Delta}_{0}\ll \left|{D}_{0}\right|\right|$. This serves for subsequent playback and constraint phases.

#### Continual learning phase(Stage S):

As the device operates, progressively degraded datasets are sequentially collected, $\left\{{D}_{1},{D}_{2}\cdots {D}_{s}\right\}$ . At stage S, the previously constructed knowledge buffer ${\Delta}_{s-1}$ is combined with the current dataset ${D}_{s}$ to directly train the prior neural network $F\left(\bullet ,{\theta }^{s-1}\right)$, yielding an optimized prediction network $F\left(\bullet ,{\theta}_{s}^{*}\right)$. During this process, parameter magnitude is constrained via EWC and parameter gradient direction via GEM. Following network training, the following two operations are performed:

1. Parameter constraint:

Deviations are penalized for parameters critical to previous tasks, while less important parameters can be updated more freely. By introducing the Fisher information for each parameter as a measure of importance, EWC can automatically regulate the magnitude of parameter updates during optimization, thereby achieving a form of“fine-grained protection.”Formally, the loss function for the current task is defined as:

2.2 $$L_{EWC} (\theta ) = L_{{{\mathrm{new}}}} (\theta ) + \lambda /2\sum\limits_{i = 1}^{k} {\sum\limits_{j = 1}^{d} {F_{j}^{(i)} } } \left( {\theta_{j} - \theta_{j}^{*(i)} } \right)^{2}$$

Here, *λ >0* denotes the regularization strength. To prevent early task Fisher information from dominating long-term learning, a decay coefficient γ ∈ (0,1] applies exponential weighting to the accumulated Fisher matrix:

2.3 $$\overline{F}^{(s)} = \gamma \cdot \overline{F}^{(s - 1)} + (1 - \gamma ) \cdot F_{{{\mathrm{new}}}}^{(s)}$$

where ${F}_{0}$ denotes the initial Fisher matrix, and ${F}_{s}$ represents the Fisher information newly computed from the current task dataset ${D}_{s}$. The term *F* in Eq. (2.1) can be replaced with the decayed Fisher matrix $\widetilde{F}$, which assigns higher importance to more recent tasks. Taking the gradient of ${L}_{EWC}$, the EWC gradient can be obtained as:

2.4 $${\mathbf{g}}_{{{\mathrm{EWC}}}} = \nabla_{{{\boldsymbol{\uptheta}}}} {\mathcal{L}}_{{{\mathrm{new}}}} ({{\boldsymbol{\uptheta}}}) + \lambda \sum\limits_{i = 0}^{s - 1} {\overline{F}^{(i)} } \odot \left( {{{\boldsymbol{\uptheta}}} - {{\boldsymbol{\uptheta}}}^{*(i)} } \right)$$

To further ensure that parameter updates for new tasks do not compromise the learning performance of old tasks, a GEM^31^ mechanism is introduced to constrain the direction of the gradient ${\mathrm{g}}_{EWC}$ output by EWC.

(2) Gradient constraint:

This mechanism prevents parameters from drifting toward directions that may degrade the performance of previous tasks during the learning of new tasks by constraining the gradient update direction. The specific procedure is as follows:

Using the samples of previous tasks stored in the knowledge buffer ${\Delta}_{S-1}$, the gradients of each previous task are computed under the current model parameters *θ*:

2.5 $${\mathbf{g}}_{i} = \nabla_{{{\boldsymbol{\uptheta}}}} {\mathcal{L}}_{i} \left( {{{\boldsymbol{\uptheta}}};{\mkern 1mu} \Delta_{s - 1}^{(i)} } \right)$$

The gradient update direction of the new task must be compatible with the gradient directions of all previous tasks. Specifically, the inner product between the new task gradient and each previous task gradient must be non-negative, which can be formulated as:

2.6 $$\widetilde{{\mathbf{g}}}^{\top} {\mathbf{g}}_{i} \ge 0,\quad \forall i \in \{ 0,1, \ldots ,s - 1\}$$

If the original gradient produced by EWC does not satisfy the above compatibility constraints, it is projected onto the feasible cone through convex quadratic programming to obtain the final gradient used for parameter updates, denoted as ${g}_{GEM}$. Under all compatibility constraints, the projected gradient $\widetilde{g}$ is obtained by minimizing its Euclidean distance to the original gradient ${g}_{EWC}$, thereby preserving as much learning information from the new task as possible:

2.7 $${\mathbf{g}}_{{{\mathrm{GEM}}}} = \arg \min_{{\widetilde{{\mathbf{g}}}}} \left\| {\widetilde{{\mathbf{g}}} - {\mathbf{g}}_{{{\mathrm{EWC}}}} } \right\|_{2}^{2} \quad {\mathrm{s}}{\mathrm{.t}}{.}\quad {\tilde{\mathbf{g}}}^{\top} {\mathbf{g}}_{i} \ge 0,\;\forall i$$

The projected gradient ${g}_{GEM}$ is then used to update the model parameters, and the final update rule is given by: ${{\boldsymbol{\uptheta}}} \leftarrow {{\boldsymbol{\uptheta}}} - \alpha {\mathbf{g}}_{{{\mathrm{GEM}}}}$ where *α > 0* denotes the learning rate.

(3) Finally, the system executes the Update of knowledge buffer to integrate representative samples from the current task for future stages.

### Uncertainty quantification with dual-dimensional spatial constraints

**Fig.2:**
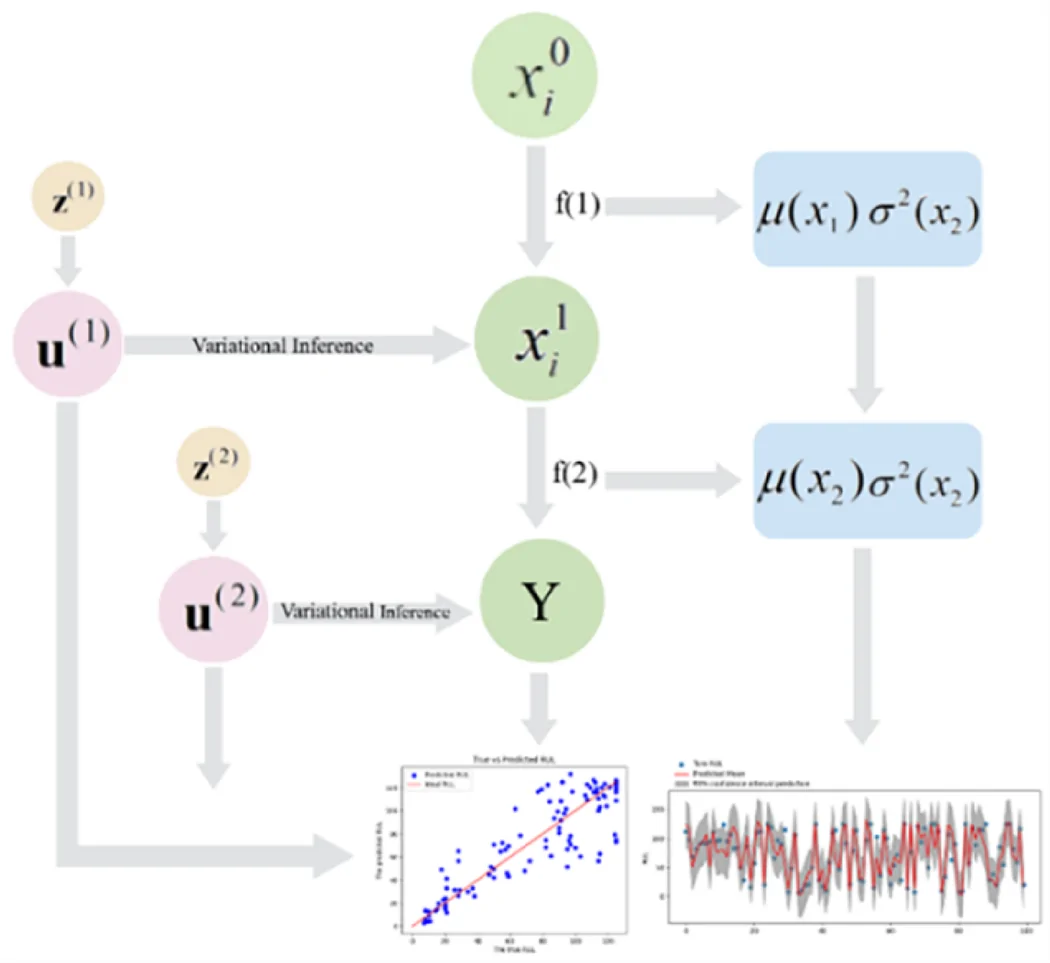
Variational deep Gaussian processes UQ framework.

To maintain the long-term effectiveness of deep degradation prediction models under cross-operating-condition learning, this study introduces a variational inducing DGP^35^ structure within the RUL prediction framework, as illustrated in Fig. 2. This approach enables the modeling of complex nonlinear degradation patterns while providing computational scalability and uncertainty quantification capabilities.

In this architecture, the output of each GP layer is used as the input to the subsequent layer, allowing the model to capture hierarchical representations of degradation features in a layer-wise manner.

Within the DGP framework, the overall marginal likelihood can be expressed as:

2.8 $$p(y\left| {x^{(0)} } \right.) = \int {\prod\limits_{l = 1}^{L} p } \left( {f^{(l)} \left| {f^{(l - 1)} } \right.} \right)p\left( {y\left| {f^{(L)} } \right.} \right)df$$

where ${f}^{(l)}$ denotes the latent function value of the GP at layer *l* . As this integral is mathematically intractable, the DGP employs variational inference for approximation and uncertainty quantification. The mean and standard deviation of the predicted values are utilised to construct a 95% confidence interval.

#### Variational inference and induced point mechanism

Traditional GP incur prohibitively high computational costs when processing large-scale rotating machinery monitoring data, with the primary bottleneck being the inversion of the covariance matrix. To address this, an induced point ${u}^{(l)}$ and induced variable ${z}^{(l)}$ are introduced to expand the probability space, constructing a joint variational distribution:

2.9 $$q(f_{1:L} ,u_{1:L} ) = \prod\limits_{l = 1}^{L} p \left( {f^{(l)} \left| {u^{(l)} ,f^{(l - 1)} } \right.} \right)q\left( {u^{(l)} } \right)$$

Here, the $q({u}^{(l)})$ is formulated as a Gaussian distribution to be optimized, approximating the true posterior. The overall log-marginal likelihood yields a variational lower bound (ELBO) via Jensen’s inequality, where:

2.10 $$\log p\left( {y\left| X \right.} \right) \ge {\mathbb{E}}_{{q(f^{1:L} )}} [\log p\left( {y\left| {f^{(L)} } \right.} \right)] - \sum\limits_{l = 1}^{L} {{\mathrm{KL}}} (q({\mathbf{u}}^{(l)} )\left\| {p({\mathbf{u}}^{(l)} } \right.))$$

Thus, the objective function becomes:

2.11 $${\mathcal{L}}_{{{\mathrm{ELBO}}}} = {\mathbb{E}}_{q(f)} [\log p(y\left| f \right.)] - {\mathrm{KL}}(q(u)\left\| {p(u)} \right.)$$

This lower bound not only substantially reduces computational complexity and suppresses deviation from the prior distribution but also ensures convergence stability under large-scale multi-scenario data.

The overall loss function is defined as follows:

2.12 $$q(f_{1:L} ,u_{1:L} ) = \prod\limits_{l = 1}^{L} p \left( {f^{(l)} \left| {u^{(l)} ,f^{(l - 1)} } \right.} \right)q\left( {u^{(l)} } \right)$$

where the second term represents the EWC regularization term, which utilizes the Fisher information matrix $\tilde{F}^{{\left( {S1} \right)}}$ to identify and penalize deviations of parameters that are critical to previous tasks. This integrated design ensures that the model remains within a shared high-performance region in the parameter space that is jointly suitable for both previous and current tasks. Consequently, the framework enables a transition from isolated uncertainty quantification to probabilistic modeling with memory preservation.

#### Optimal distribution of induced variables

During continual learning, the representational capability of the inducing variables is highly dependent on the mapping defined by the kernel function parameters *θ*. Without appropriate constraints, data from new operating conditions may force *θ* to shift drastically, which can distort the kernel matrices ${K}_{uu}$ and ${K}_{uf}$. This distortion may further cause the distribution of the inducing variables to collapse near the boundary between previous and new tasks.

By optimizing the induced variables at different levels ${\mathbf{u}}^{(l)}$, the optimal Gaussian distribution form can be analytically derived. The general-layer induced variable distribution is:

2.13 $$q^{*} (u^{(l)} ) \propto p(u^{(l)} )\exp \left\{ {{\mathbb{E}}_{{q(f^{(l - 1)} )}} \left[ {\log p(f^{(l)} \left| {u^{(l)} ,f^{(l - 1)} } \right.)} \right]} \right\}$$

Intermediate-layer induced variable distribution:

2.14 $$q^{*} (u^{(l)} ) \propto p(u^{(l)} )\exp \left\{ {{\mathbb{E}}_{{q(f^{(l)} ,{\kern 1pt} f^{(l - 1)} )}} \left[ {\log p(f^{(l)} \left| {u^{(l)} ,f^{(l - 1)} } \right.)} \right]} \right\},l \in \{ 2, \ldots ,L - 1\}$$

First-layer induced variable distribution:

2.15 $$q^{*} (u^{(1)} ) \propto p(u^{(1)} )\exp \left\{ {{\mathbb{E}}_{{q(f^{(1)} )}} \left[ {\log p(f^{(1)} \left| {u^{(1)} ,X} \right.)} \right]} \right\}$$

As the aforementioned optimization objectives are all quadratic forms concerning ${u}^{(l)}$ , the optimal solution must be a Gaussian distribution: ${q}^{*}\left({u}^{(l)}\right)=N\left({m}^{(l)},{S}^{(l)}\right)$ The mean ${m}^{\left(l\right)}$ and covariance ${s}^{\left(l\right)}$ are computed from the kernel matrices parameterized by *θ*. During training, EWC penalizes large variations in *θ*, thereby indirectly constraining the search space of ${m}^{\left(l\right)}$ and ${s}^{\left(l\right)}$.This implies that while the model updates the variational parameters, the stability of the model weights *θ* is preserved. As a result, degradation patterns can be progressively propagated across multiple GP layers, enabling effective and robust feature representation.

#### Uncertainty quantification

After optimizing the ELBO, the deviation between the variational distribution and the prior is suppressed. The DGP approximates the true posterior: *p(u|X,y)*. During prediction, for any test input $x^{*}$, the mean and variance are estimated as:

2.16 $$\mu (x_{*} ) = {\mathbb{E}}_{{q(f^{(L)} )}} [f^{(L)} (x_{*} )],\quad \sigma^{2} (x_{*} ) = {\mathrm{Var}}_{{q(f^{(L)} )}} [f^{(L)} (x_{*} )]$$

Furthermore, the 95% confidence interval is constructed:

2.17 $${\mathrm{UB}} = \mu (x_{*} ) + z_{\alpha /2} {\mkern 1mu} \sigma (x_{*} )$$

2.18 $${\mathrm{LB}} = \mu (x_{*} ) - z_{\alpha /2} {\mkern 1mu} \sigma (x_{*} ),z_{{\alpha {/}2}} = 1.96$$

Consequently, the model not only predicts RUL but also provides its confidence interval, facilitating robust life estimation under complex operating conditions.

## Experimental verification

In this section, the effectiveness of the proposed CL framework is validated using the accelerated bearing degradation dataset from Xi’an Jiaotong University^36^ and wind farm datasets. Additionally, ablation studies are conducted, and the proposed framework is compared with state-of-the-art methods.

### Data description and experimental setup

**Fig.3:**
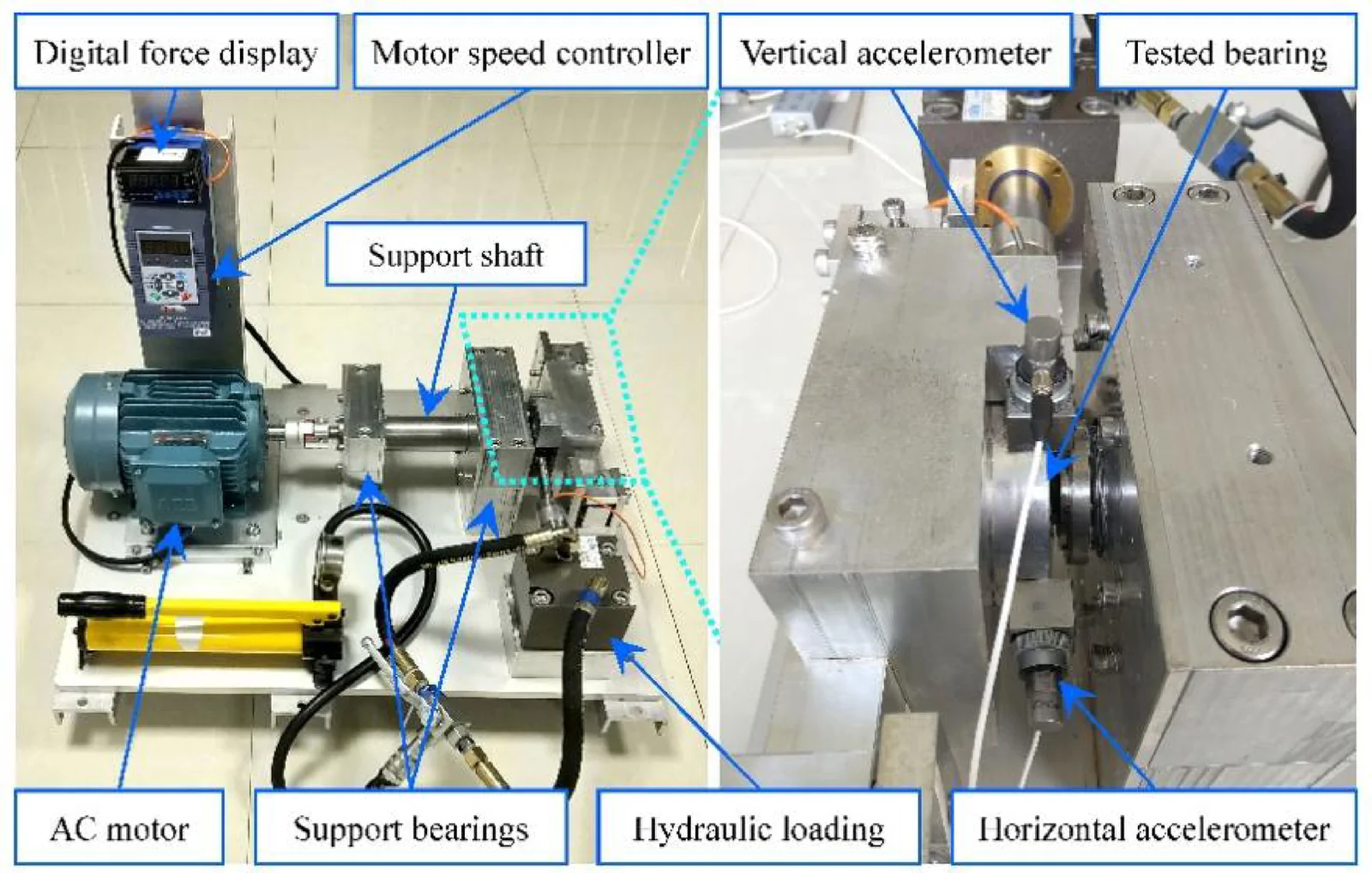
Rolling element bearing testbed.

Xi’an Jiao tong University Bearing Dataset. As illustrated in Fig. 3, accelerated degradation experiments were conducted on a rolling bearing test rig. Data acquisition was performed on the LDKUER204 bearing test rig, driven by an alternating current (AC) induction motor. The test rig configured three distinct operating conditions for accelerated degradation testing of rolling element bearings, with four bearings tested under each condition. Operating conditions comprised: (1) 2100 rpm (35 Hz) and 12 KN; (2) 2250 rpm (37.5 Hz) and 11 KN; (3)2400 rpm (40 Hz) and 10 KN. Two PCB352C33 accelerometers sampled the vertical and horizontal vibration signals of the tested bearings at a sampling frequency of 25.6 kHz. Each sampling cycle recorded 32,768 data points (equivalent to 1.28 s), with a sampling period of 1 min. As shown in Table 1.

**Table 1:** Operating conditions of tested bearings.

Operating conditions	Rotating speed	Radial force	Bearings
Condition 1	2100 rpm	12KN	Bearing1_1-Bearing1_4
Condition 2	2250 rpm	11KN	Bearing2_1-Bearing2_4
Condition 3	2400 rpm	10KN	Bearing3_1-Bearing3_4

**Fig.4:**
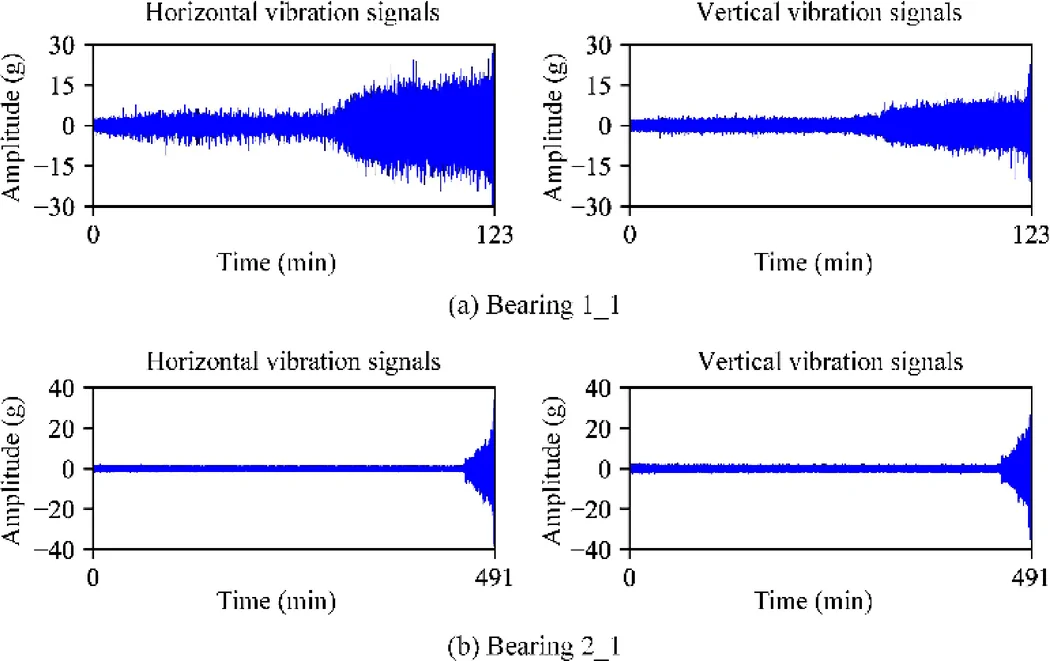
Horizontal and vertical vibration signals of bearings (**a**) Horizontal and vertical signals of bearing 1–1 (**b**) Horizontal and vertical signals of bearing 2–1.

**Fig.5:**
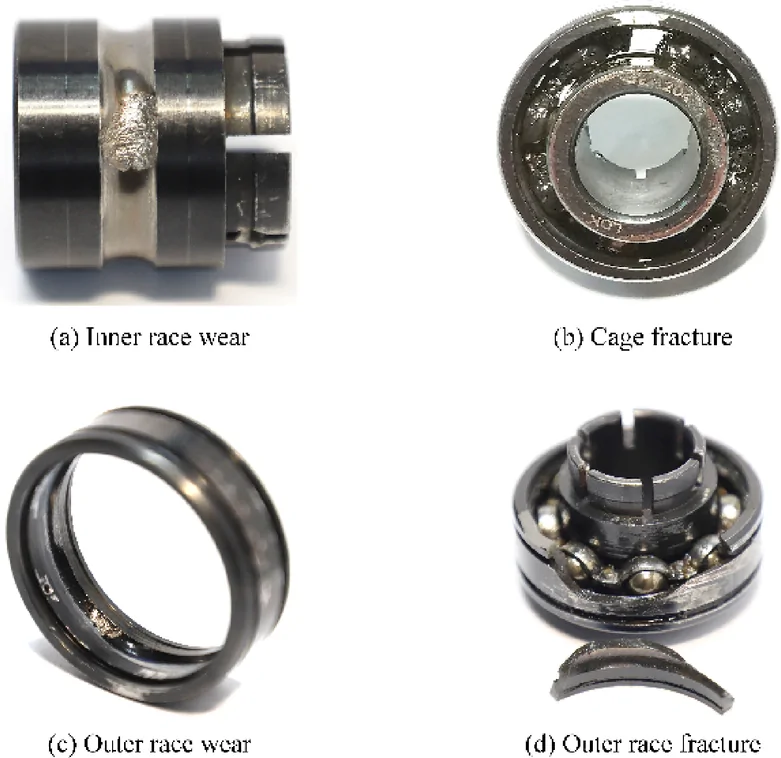
Images of degraded bearings (**a**) Inner ring wear (**b**) Bearing cage fracture (**c**) Outer ring wear (**d**) Outer ring cage surface.

Acceleration degradation tests were conducted on 12 test bearings under three distinct operating conditions. Bearing failure types are summarized in Table 2. Figure 4 illustrates the vibration signal of a specific bearing. Figure 5 shows an image of a degraded bearing. A CL scenario was established, designs three cross-operating-condition experiments: three bearing datasets are selected from Operating Condition 1, 2, and 3, respectively, and used as learning tasks for Stage 1, Stage 2, and Stage 3. Upon the completion of learning at each stage, bearing data from prior stages is adopted to evaluate the forgetting behavior of the proposed framework. To systematically evaluate the proposed method, the experiments are organized into three groups:

**Table 2:** Failure types and life of the tested bearings.

Operating condition	Bearing dataset	Number of files	Bearing lifetime	Fault element
Condition 1 (35 Hz/12kN)	Bearing 1_1	123	2 h 3 min	Outer race
	Bearing 1_2	161	2 h 41 min	Outer race
	Bearing 1_3	158	2 h 38 min	Outer race
	Bearing 1_4	122	2 h 2 min	Cage
Condition 2 (37.5 Hz/11kN)	Bearing 2_1	491	8 h 11 min	Inner race
	Bearing 2_2	161	2 h 41 min	Outer race
	Bearing 2_3	533	8 h 53 min	Cage
	Bearing 2_4	42	42 min	Outer race
Condition 3 (40 Hz/10kN)	Bearing 3_1	2638	42 h 18 min	Outer race
	Bearing 3_2	2496	41 h 36 min	Inner race, ball, cage and outer race
	Bearing 3_3	371	6 h 11 min	Inner race
	Bearing 3_4	1515	25 h 15 min	Inner race

1. Group 1: validates the overall performance of the proposed framework;
2. Group 2: conducts ablation studies to analyze the effectiveness of the dual-dimensional constraint mechanism;
3. Group 3: compares dual-dimensional continual learning strategy against state-of-the-art (SOTA) continual learning approaches to assess its generalization capability and resistance to catastrophic forgetting. Details are presented in Table 3.

**Table 3:** Experimental designs for continual learning scenarios.

	Stage1	Stage2	Stage3
Group1	Bearing1-4	Bearing3-3	Bearing2-3
Group2	Bearing3-4	Bearing1-1	Bearing2-1
Group3	Bearing1-1	Bearing2-4	Bearing3-2

Wind Turbine Gearbox Dataset. To evaluate the applicability of the proposed method in practical scenarios, experimental data from the Hangjinqi Wind Farm in Inner Mongolia was utilized. The wind farm comprises 31 turbines, with monitoring data from three identical turbines selected for experimentation. Details are presented in Table 4. The experimental sequence is shown in Table 5.

**Table 4:** Experimental group settings of wind turbine gearboxes.

Continual learning	Wind turbine gearbox
Step 1	3
Step 2	6
Step 3	11

**Table 5:** Technical specifications of the wind turbine gearbox.

Turbine type		3	6	11
Wind turbine gearbox	Wind Class（KW）	IEC I/S	IEC Ⅱ	IEC Ⅱ
	Operating Ambient Temperature (℃)	Operating Ambient Temperature-25~+50，Low Temperature Type-45~+50		
Rotor	Number of Blades	3		
	Rotor Diameter（m）	70	77	82
Gearbox	Structure Type	2-stage planetary + 1-stage parallel shaft gear		

The structural configuration and technical specifications of the Wind Turbine Gearbox transmission system are detailed in Table 5. The dataset comprises vibration signal characteristics from 12 measurement points: gearbox secondary planetary radial, upper frame (vertical shaft), main shaft rear main bearing radial, and main shaft front main bearing radial, among others. The Wind Turbine Gearbox operated continuously until failure occurred. As failures typically manifest during the later operational phase—a critical period for maintenance and repairs—only late-stage monitoring data was selected for RUL predictive analysis. The dataset was partitioned into training and test sets for model training and evaluation. All data underwent Min–Max regularization to the range [− 1,1].

All experiments were conducted on a computer configured with an Intel® Core™ i9-14900 K processor (3.20 GHz), 32 GB RAM, and an NVIDIA GeForce RTX 4070 Ti graphics card.

### Evaluation metrics

In this paper, the root mean square error (RMSE)^37^ is employed to evaluate the proposed framework’s RUL prediction performance.

Additionally, the average forgetting rate (AF) is designed to measure the extent to which the deep RUL prediction network forgets learned knowledge. During continual learning, the AF value is calculated as follows:

3.1 $$AF_{T} = \left( {\frac{1}{T - 1}} \right)\sum\limits_{i = 1}^{T - 1} {F_{i}^{T} }$$

3.2 $$F_{i}^{T} = \frac{{R_{i}^{Tfinal} - R_{i}^{Tbest} }}{{R_{i}^{Tbest} }}$$

Here,$A{F}_{T}$ denotes the average forgetting rate prior to phase *T* ;${f}_{i}^{T}$ characterises the memory decay of the network for the *i* th learning task after completing phase *T* ;${R}_{i}^{Tbest}$ represents the absolute error computed by the network for the *i* th training dataset during phase *T* . When $A{F}_{T}$ exhibits a low value, it indicates that the model demonstrates minimal forgetting of acquired knowledge during continual learning, thereby maintaining superior stability and memory retention capabilities. In subsequent experimental sections, each experiment was independently run 5 times, with the average value taken to mitigate random fluctuations. Furthermore, all evaluation metrics were calculated based on the full lifetime data of each bearing.

### Configuration of the proposed framework

To ensure the robustness and reproducibility of the proposed framework, the key hyperparameters were determined using a grid search strategy on the training and validation sets. Instead of randomly selecting parameter values, predefined search ranges were specified for each critical parameter, and the RMSE was used as the evaluation metric for systematic comparison.

1. *Network architecture parameters* For the number of DGP layers *L* and the number of hidden neurons *H*, balanced experiments were conducted within the ranges $\left\{1,2,3,4\right\}$ and $\left\{8,10,16,32\right\}$, respectively. The results indicate that when *L=2* and *H=10*, the model maintains sufficient nonlinear representation capability while effectively preventing overfitting.
2. *Continual learning regularization* The regularization coefficient *λ* was searched within the interval $\left[1,100\right]$ using logarithmic steps. Comparative experiments show that *λ =30* provides the best balance between resistance to catastrophic forgetting and the learning ability for new knowledge.
3. *Experience replay ratio* The replay ratio *R* was evaluated within the range of 10%–50%. The final value *R=0.3* was selected to balance computational overhead and long-term memory retention.

All experiments were independently repeated five times with the same initial learning rate ${L}_{r}=1\times {10}^{-3}$ to ensure statistical reliability of the results. The detailed parameter configurations are summarized in Table 6.

**Table 6:** Hyperparameter configurations of the proposed framework.

Hyperparameter	Size	Hyperparameter	Size
DGP-layer	2	Batch-size	32
Hidden-dim	10	Lambda	30
Num-inducing	128	Replay-ratio	0.3
Reference loss	$1\times {10}^{-3}$		

### Analysis of remaining useful life prediction results

This section demonstrates the performance of the proposed dual-dimensional constrained continual learning uncertainty quantification framework in RUL prediction tasks. The framework demonstrates excellent prediction accuracy measured by RMSE on both the Xi’an Jiao tong University bearing dataset and the wind farm turbine dataset. It maintains a low AF throughout the multi-stage incremental learning process, validating its robustness and knowledge retention capability across operating conditions.

**Fig.6:**
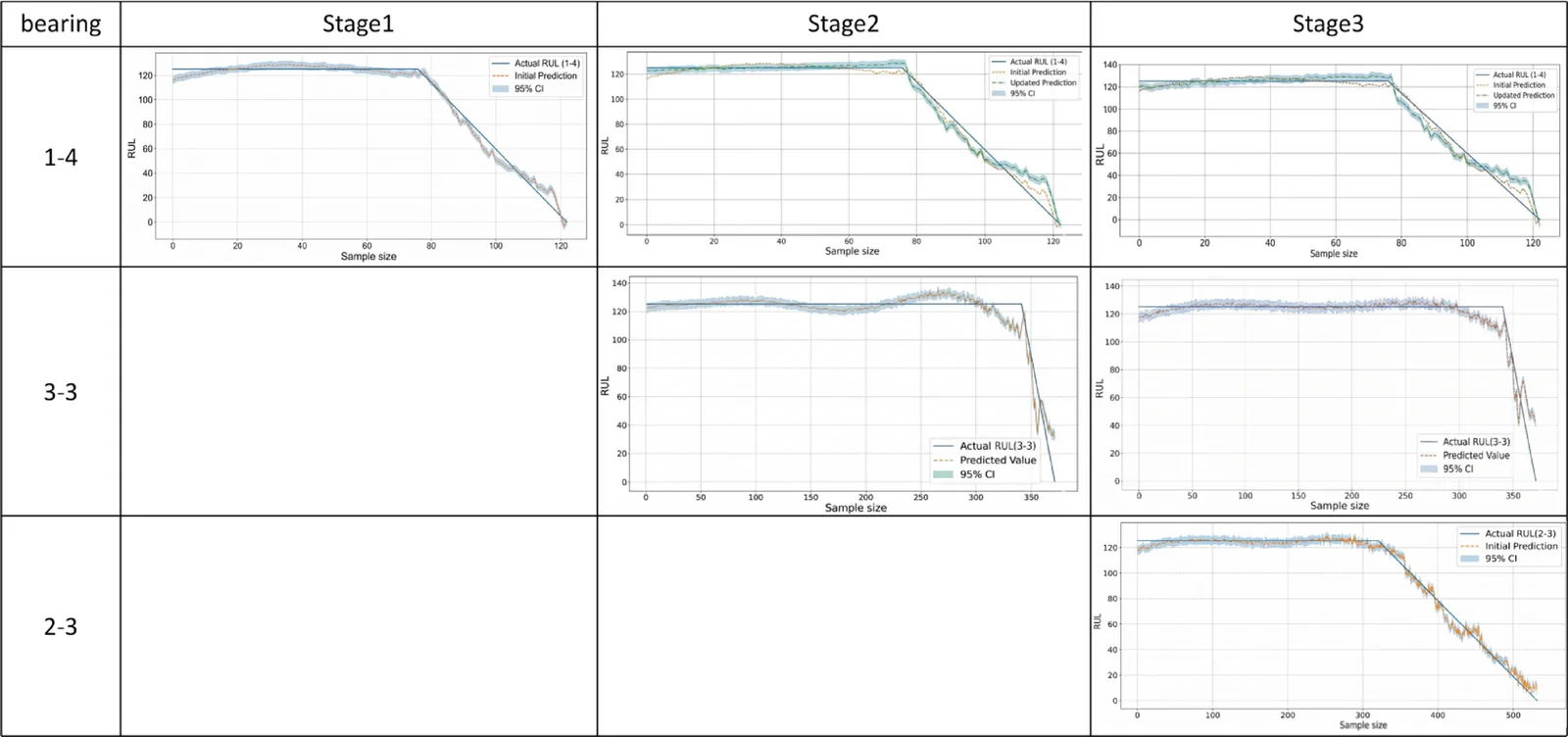
Prediction results of bearing RUL at different continual learning stages.

Figure 6 depicts the dynamic prediction curves for the RUL of each bearing as the model continuously incorporates data from different operating conditions. Table 7 presents the corresponding RMSE and AF metrics. It can be observed that the prediction curves exhibit overall smoothness, closely aligning with the actual degradation trends while maintaining stable error levels throughout the different task stages. Quantitatively, the RMSE values for the initial task (Bearing 1–4) across the three continual learning stages are 4.3, 6.1, and 7.1, respectively. For the sequentially added tasks, the model achieves an RMSE of 6.62 on Bearing 3–3 at Stage 2, and maintains high accuracy at Stage 3 with RMSEs of 7.50 (Bearing 3–3) and 3.66 (Bearing 2–3). This demonstrates the model’s sustained high predictive accuracy following multi-task transfer. Concurrently, the AF metrics across the three stages are 0, 0.53, and 0.39, respectively. Stage 1 exhibits negligible forgetting, while the subsequent stages demonstrate significantly lower forgetting rates compared to traditional neural networks (which typically yield AF > 1). This indicates that the framework not only rapidly adapts to and accurately predicts the current operating condition’s life trend, but also maintains robust predictive performance for prior operating conditions even when significant equipment degradation occurs, demonstrating robust continual learning capabilities.

**Table 7:** Prediction results of bearing RUL at different continual learning stages.

Bearing		Stage 1	Stage 2	Stage 3
RMSE	Bearing1-4	4.3 (± 0.04)	6.1 (± 0.03)	7.1 (± 0.04)
	Bearing3-3		6.62 (± 0.02)	7.50 (± 0.02)
	Bearing2-3			3.66 (± 0.04)
AF		0	0.53 (± 0.02)	0.39(± 0.03)

As tasks transition from Bearing1-4 to Bearing2-3 and Bearing3-3, the prediction curves remain stable with limited fluctuations, demonstrating strong generalization across operating conditions. The emergence of high-variance regions, together with corresponding AF variations, validates the effectiveness of uncertainty quantification in jointly supporting risk awareness and memory retention.

**Fig.7:**
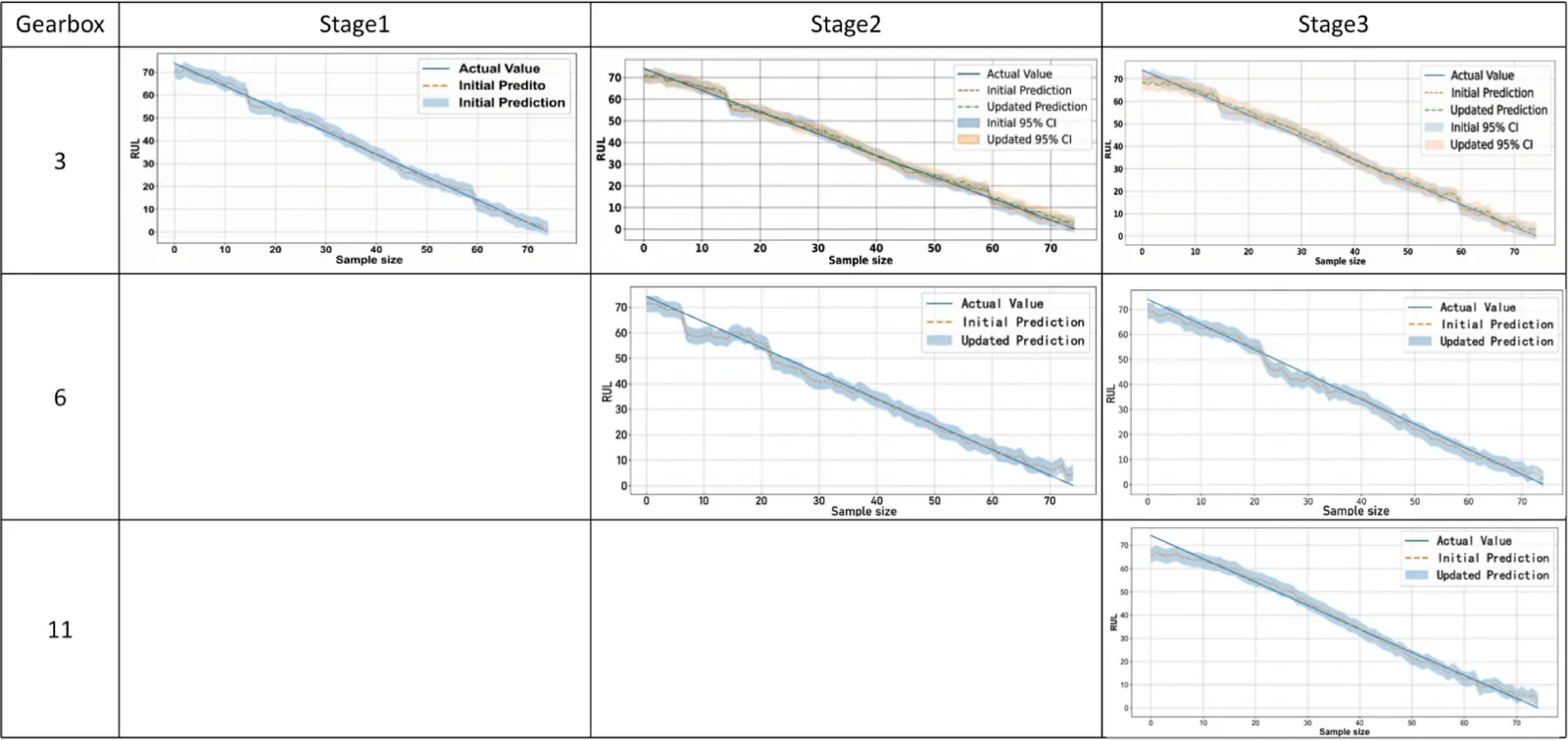
Prediction results of RUL for each Wind Turbine Gearbox under different continual learning stages.

To further demonstrate the continual learning capability of the proposed uncertainty-aware and dual-constraint continual learning framework for cross-device RUL prediction, monitoring data from three Wind Turbine Gearbox of the same model were selected for evaluation. The experimental results are illustrated in Fig. 7, and the corresponding quantitative metrics are summarized in Table 8.

**Table 8:** Prediction results of RUL for each wind turbine gearbox under different continual learning stages.

Wind turbine gearbox		Stage 1	Stage 2	Stage 3
RMSE	Gearbox 3	1.36 (± 0.02)	1.58 (± 0.01)	1.85 (± 0.02)
	Gearbox 6		2.60 (± 0.02)	2.12 (± 0.03)
	Gearbox 11			2.22 (± 0.02)
AF		0	0.16 (± 0.01)	0.18(± 0.02)

In Domain 1, the model achieved an initial RMSE of 1.36 on wind turbine gearbox 3. When the training proceeded to Domain 2, the model was updated using data from wind turbine gearbox 6, resulting in an RMSE of 2.60 on the new task, while the RMSE on the previous task (wind turbine gearbox 3) increased slightly to 1.58, indicating the emergence of mild performance degradation. After entering Domain 3, the model was further trained on wind turbine gearbox 11, achieving an RMSE of 2.22 on the new task. The retrospective evaluation on historical tasks shows that the RMSE on wind turbine gearbox 3 became 1.85, while the RMSE on wind turbine gearbox 6 improved to 2.12, which is lower than its value 2.60 in Domain 2. This phenomenon indicates that the model not only mitigates catastrophic forgetting but also performs knowledge refinement and positive transfer toward earlier tasks.

Meanwhile, the predictive uncertainty produced by the DGP evolves dynamically during task progression. For wind turbine gearbox 3, the predictive uncertainty gradually increases as new tasks are introduced, reflecting the decreasing confidence of the model on earlier tasks. In contrast, for wind turbine gearbox 6, the predictive uncertainty decreases concurrently with the convergence of RMSE, suggesting that the model improves its reliability in representing this device with the assistance of newly observed data.

### Ablation experiments

**Fig.8:**
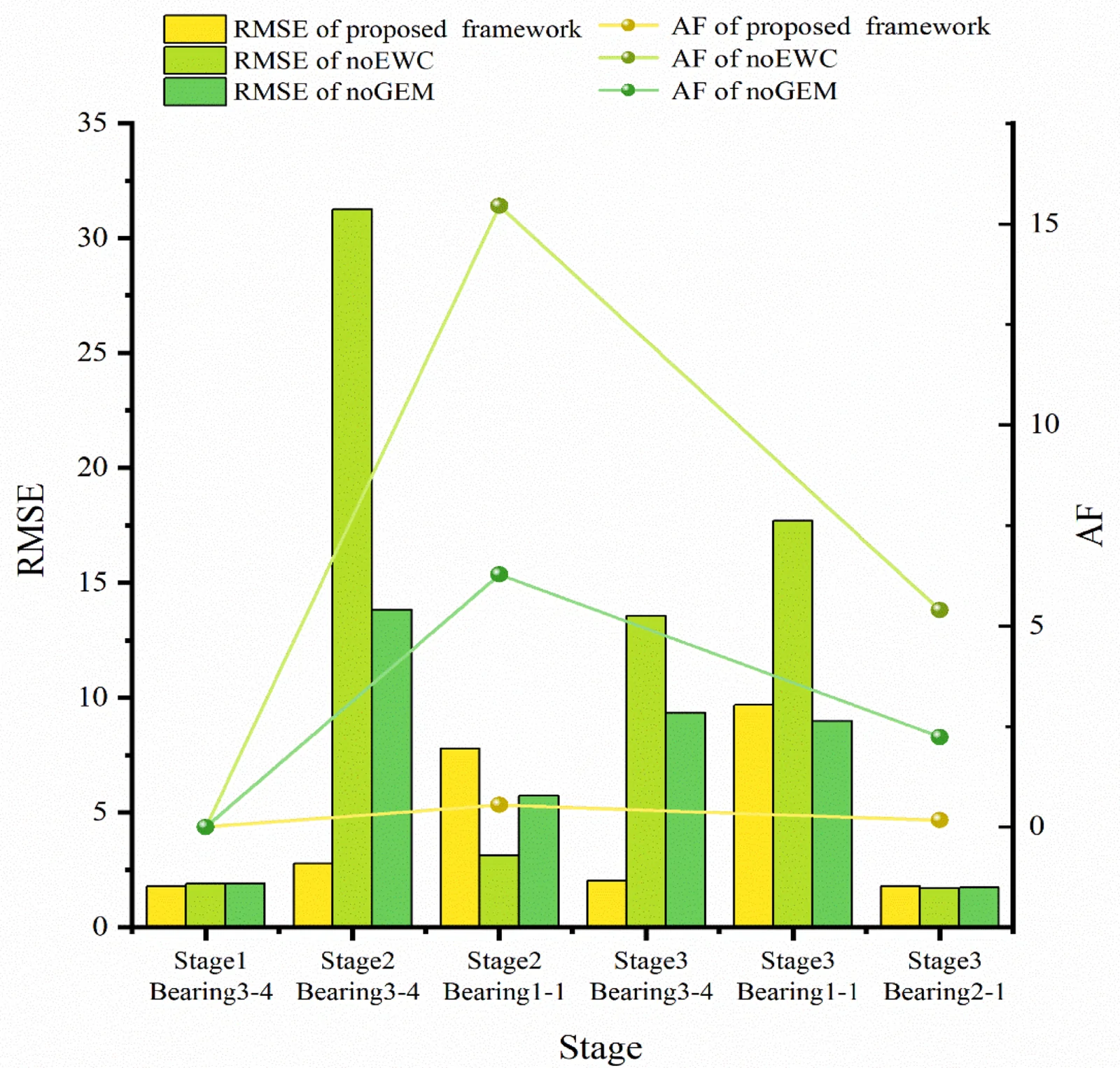
Ablation experiments using bearing datasets to analyze the effect of continual learning.

To comprehensively evaluate the contributions of key components in the proposed dual-constraint continual learning framework, systematic ablation experiments were conducted. In these experiments, each mechanism—EWC and GEM—was sequentially removed while keeping the remaining components unchanged, in order to examine their respective impacts on prediction accuracy and resistance to catastrophic forgetting. As illustrated in Fig. 8.

Experiments were performed on three bearing datasets from the Xi’an Jiao tong University Bearing Dataset: Bearing3-4 (initial task), Bearing1-1 (Condition 2), and Bearing2-1 (Condition 3). The evaluation metrics include RMSE and AF, and the results are summarized in Table 9.

**Table 9:** Ablation experiments using bearing datasets to analyze the effect of continual learning.

Methods			Stage						
EWC	GEM	stage1 3–4	stage2 3–4	stage2 1–1	stage3 3–4	stage3 1–1	stage3 2–1	Stage2	Stage3
		RMSE	RMSE	RMSE	RMSE	RMSE	RMSE	AF	AF
✓	✓	**1.8** (± 0.04)	**2.79** (± 0.04)	7.8 (± 0.09)	**2.04** (± 0.04)	9.7 (± 0.11)	1.8 (± 0.03)	**0.55** (± 0.02)	**0.17** (± 0.02)
×	✓	1.9 (± 0.05)	31.26 (± 0.35)	**3.13** (± 0.05)	13.57 (± 0.14)	17.71 (± 0.23)	**1.72** (± 0.03)	15.45 (± 0.20)	5.39 (± 0.10)
✓	×	1.91 (± 0.05)	13.82 (± 0.18)	5.73 (± 0.07)	9.34 (± 0.12)	**8.98** (± 0.12)	1.74 (± 0.03)	6.28 (± 0.10)	2.24 (± 0.05)

Significant values are in bold.

#### Analysis of the EWC mechanism’s effectiveness

When the EWC mechanism is removed, the proposed DGP-based model exhibits a dramatic increase in RMSE on the initial task (Bearing3-4), rising from 1.90 to 31.26, as shown in Fig. 8, while the AF increases to 5.39. This result indicates that the model suffers severe catastrophic forgetting of the degradation patterns learned from the initial task when adapting to a new operating condition (Bearing1-1).

EWC estimates the importance of network parameters using the Fisher Information Matrix and applies a quadratic regularization penalty to parameters with high importance during subsequent task optimization. This mechanism effectively constrains large deviations in critical parameters. The observed results confirm that EWC plays a crucial role in preserving historical knowledge during multi-stage continual learning, thereby serving as a key mechanism for maintaining model memory stability.

#### Analysis of the GEM mechanism’s role

When the GEM mechanism is removed, as illustrated in Fig. 8, the AF increases significantly to 2.24, indicating a notable increase in forgetting. This phenomenon suggests that GEM constrains the compatibility between the update direction of new task parameters and historical knowledge in the gradient space. Specifically, during optimization, GEM projects the gradient of the current task so that its inner product with the gradients of previous tasks remains non-negative.

Importantly, GEM does not directly rely on historical data replay nor impose explicit regularization on model parameters. Instead, it introduces an implicit gradient correction mechanism that prevents the optimization trajectory from conflicting with the learning directions of previous tasks, thereby mitigating catastrophic forgetting. Furthermore, when GEM is combined with EWC, the gradient-level compatibility provided by GEM complements the Fisher-information-based parameter preservation of EWC. This synergy not only reduces knowledge degradation during long-term learning but also improves the model’s generalization capability and adaptability in multi-task transfer scenarios.

### Comparison with state-of-the-art methods

To ensure fair and scenario-adaptive comparisons among different continual learning methods, this section adopts TCN as the backbone neural architecture. TCN are selected primarily due to their inherent advantages in modeling sequential data. This design eliminates performance bias caused by differences in network architectures and allows the comparison to focus on the intrinsic effectiveness of various continual learning strategies in mitigating forgetting.

In this section, five state-of-the-art continual learning methods for RUL prediction are implemented for comparison. Synaptic Intelligence (SI) adopts a regularization-based strategy to estimate the importance of model parameters and penalize their changes during subsequent tasks, thereby preserving knowledge from previous tasks^38^. Learning Without Forgetting (LWF) utilizes a knowledge distillation mechanism to retain information learned from earlier tasks^39^. PONNET introduces a dynamic network architecture that adapts the model structure as new tasks are encountered^40^. iCaRL + CSCCT integrates an experience replay mechanism with category-specific contrastive learning to maintain historical knowledge representations^41^. Co^2^L incorporates contrastive learning into a continual learning framework to improve feature representation and mitigate catastrophic forgetting^42^.

**Fig.9:**
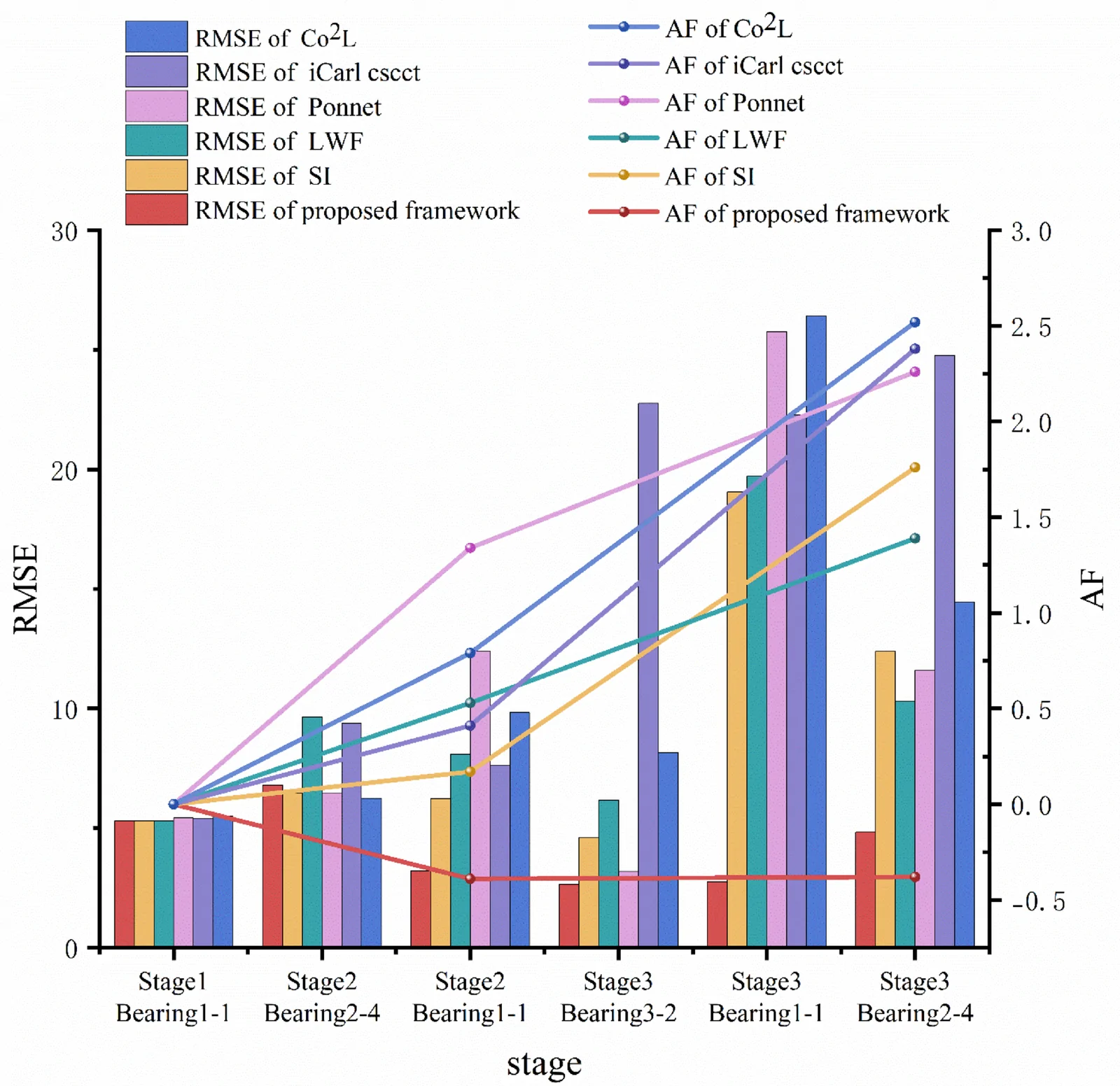
Quantitative results of different methods on bearing datasets.

**Fig.10:**
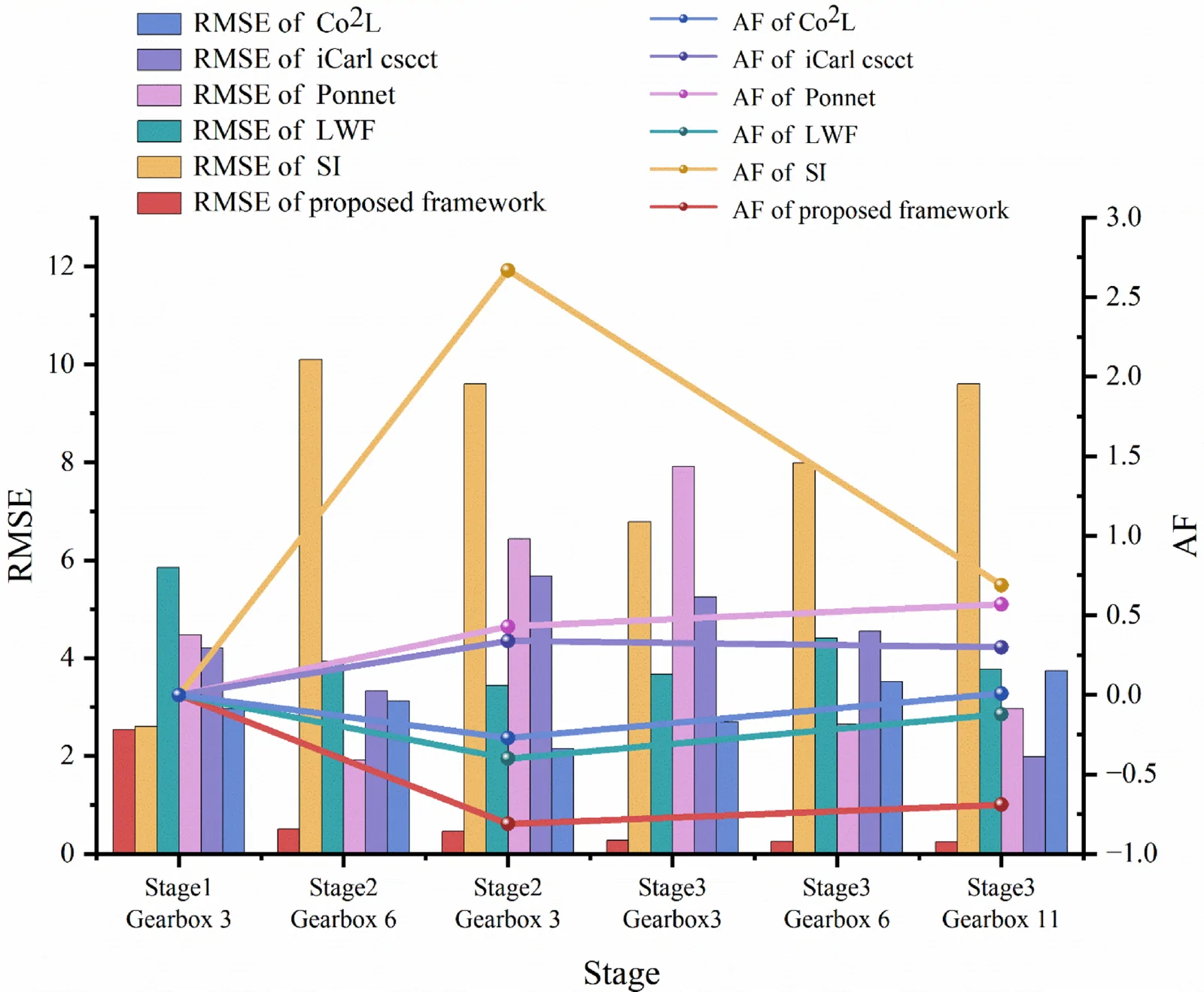
Quantitative results of different methods on wind turbine gearbox datasets.

To further validate the proposed framework across diverse devices and operating conditions, experiments are conducted using both the Xi’an Jiao tong University bearing dataset and a cross-device Wind Turbine Gearbox Dataset. The proposed method is compared with five advanced continual learning approaches (SI, LWF, PONNET, iCaRL + CSCCT, and Co^2^L). The results of these experiments are illustrated in Fig. 9 and Fig. 10, based on which the following analyses are conducted.

Firstly, as shown in Table 10 for the Xi’an Jiao tong University bearing dataset, most comparative methods exhibit significant catastrophic forgetting as the learning tasks progress from Stage 1 (Bearing 1–1) → Stage 2 (Bearing 2–4) → Stage 3 (Bearing 3–2). In particular, during the third stage, the AF values of Co^2^L, iCaRL + CSCCT, and PONNET increase rapidly, accompanied by a progressive rise in RMSE. In the Bearing 3–2 stage, while iCaRL + CSCCT experiences severe performance degradation with an RMSE exceeding 20, other baseline methods also exhibit noticeably elevated errors compared to the proposed framework. These results indicate that, under scenarios with substantial cross-operating-condition distribution shifts, methods relying solely on feature consistency constraints, hard sample reconstruction, or structured replay struggle to effectively preserve long-term knowledge.

**Table 10:** Quantitative results and parameters of different methods on bearing datasets.

Methods	Stage						Stage2 AF	Stage3 AF
	Stage1	Stage2	Stage2	Stage3	Stage3	Stage3		
	Bearing 1–1 RMSE	Bearing 1–1 RMSE	Bearing 2–4 RMSE	Bearing 1–1 RMSE	Bearing 2–4 RMSE	Bearing 3–2 RMSE		
Proposed framework	**5.29** (± 0.04)	6.79 (± 0.05)	**3.22** (± 0.03)	**2.76** (± 0.02)	**4.83** (± 0.04)	**2.65** (± 0.02)	**− 0.39** (± 0.02)	**− 0.38** (± 0.02)
SI	**5.29** (± 0.05)	6.46 (± 0.06)	6.24 (± 0.07)	19.06 (± 0.15)	12.4 (± 0.12)	4.61 (± 0.05)	0.17 (± 0.03)	1.76 (± 0.04)
Lwf	**5.29** (± 0.05)	9.65 (± 0.09)	8.09 (± 0.08)	19.71 (± 0.18)	10.31 (± 0.10)	6.17 (± 0.12)	0.52 (± 0.04)	1.39 (± 0.03)
Ponnet	5.43 (± 0.06)	6.48 (± 0.07)	12.39 (± 0.15)	25.76 (± 0.25)	11.6 (± 0.10)	3.2 (± 0.02)	1.34 (± 0.05)	2.26 (± 0.05)
iCaRL CSCCT	5.4 (± 0.06)	12.4 (± 0.12)	10.31 (± 0.12)	11.6 (± 0.12)	24.77 (± 0.22)	22.75 (± 0.32)	0.41 (± 0.04)	2.38 (± 0.06)
Co^2^L	5.49 (± 0.06)	**6.24** (± 0.06)	9.83 (± 0.10)	26.42 (± 0.25)	14.44 (± 0.13)	8.16 (± 0.02)	0.79 (± 0.04)	2.52 (± 0.06)

Significant values are in bold.

Although SI and LWF maintain a certain level of stability during the early stages, their designs impose only soft constraints on parameter variations or output distributions. Consequently, they lack a robust structural memory mechanism to address deep feature distribution drift caused by operating condition transitions. In addition, these approaches do not employ explicit replay samples as anchors for historical tasks, nor do they introduce gradient conflict suppression strategies to regulate the optimization direction between new and old tasks. As a result, during cross-stage cumulative learning, the model gradually prioritizes the current task, leading to noticeable degradation in the performance of earlier tasks during the second and third stages. Consistent with this behavior, both the AF metrics and the RMSE on historical tasks for SI and LWF show a continuous upward trend in the subsequent stages. This observation indicates that relying solely on parameter regularization or distillation-based losses is insufficient to address large distribution shifts across operating conditions.

Secondly, the cross-device experiments on Wind Turbine Gearbox, as illustrated in Fig. 10, further validate the practical applicability of the proposed method. Monitoring data from three identical wind turbine gearbox models across different stages (stage3, stage6, and stage11) were used for evaluation. The results show that several methods exhibit negative transfer, where the prediction error decreases when the model is transferred to new devices. Specifically, as shown in Table 11, the proposed method and LWF consistently demonstrate negative AF values, while Co^2^L only exhibits this negative trend in the early stage. However, their negative transfer behaviors differ significantly. It is important to note that comparing AF values alone is insufficient to determine the superiority of a method. Only when a negative AF is accompanied by a significant reduction in RMSE can it be considered a truly beneficial negative transfer, meaning that learning from new tasks improves the model’s performance on previous tasks.

**Table 11:** Quantitative parameters of different methods on wind turbine gearbox.

Methods	Stage						Stage2 AF	Stage3 AF
	Stage1	Stage2	Stage2	Stage3	Stage3	Stage3		
	Gearbox 3 RMSE	Gearbox 6 RMSE	Gearbox 3 RMSE	Gearbox 3 RMSE	Gearbox 6 RMSE	Gearbox 11 RMSE		
Proposed Framework	**2.54** (± 0.03)	**0.51** (± 0.02)	**0.46** (± 0.02)	**0.28** (± 0.01)	**0.26** (± 0.02)	**0.25** (± 0.02)	**− 0.81** (± 0.04)	**− 0.69** (± 0.04)
SI	2.61 (± 0.05)	10.1 (± 0.15)	9.6 (± 0.12)	6.79 (± 0.10)	7.99 (± 0.08)	9.6 (± 0.12)	2.67 (± 0.07)	0.69 (± 0.03)
Lwf	5.85 (± 0.08)	3.94 (± 0.05)	3.45 (± 0.04)	3.67 (± 0.05)	4.41 (± 0.06)	3.77 (± 0.05)	− 0.4 (± 0.04)	− 0.12 (± 0.02)
Ponnet	4.48 (± 0.06)	1.92 (± 0.03)	6.44 (± 0.08)	7.92 (± 0.10)	2.65 (± 0.03)	2.97 (± 0.04)	0.43 (± 0.03)	0.57 (± 0.03)
iCaRL CSCCT	4.21 (± 0.06)	3.33 (± 0.04)	5.68 (± 0.07)	5.25 (± 0.07)	4.55 (± 0.06)	1.98 (± 0.03)	0.34 (± 0.03)	0.3 (± 0.04)
Co^2^L	2.97 (± 0.04)	3.13 (± 0.04)	2.15 (± 0.03)	2.7 (± 0.03)	3.53 (± 0.05)	3.75 (± 0.05)	− 0.27 (± 0.03)	0.01 (± 0.03)

Bold values represent the optimal results in each evaluation stage.

Before proceeding to the comparative analysis, it is important to clarify the apparent discrepancy in AF signs between Tables 8 and 11. Note that this difference arises from their distinct validation setups. Table 8 evaluates the inherent stability of the integrated DGP framework yielding a low positive AF, whereas Table 11 benchmarks the dual-dimensional CL strategy against SOTA methods on a shared TCN backbone, where a negative AF indicates beneficial backward transfer.

Therefore, the following analysis evaluates transfer quality by jointly considering AF and RMSE. The combined trends of these two metrics indicate that the proposed continual learning framework achieves the lowest RMSE across all stages, while also exhibiting the most pronounced negative AF among all compared methods. This result suggests that, after transferring to new Wind Turbine Gearbox, the proposed method not only preserves knowledge from earlier tasks but also significantly improves overall prediction accuracy. Such behavior represents the most desirable form of beneficial negative transfer. In contrast, although Co^2^L and LWF also show negative AF values, their RMSE reductions are less significant than those observed in the proposed method. In other words, their negative transfer effects are either weaker in magnitude or accompanied by relatively higher absolute prediction errors, making them less stable and less valuable from an engineering perspective.

A comparison across the two datasets further reveals that the proposed framework maintains a consistently stable advantage in both tasks. This demonstrates that the proposed method is effective not only in cross-operating-condition learning scenarios, but also under cross-device distribution shifts. Specifically, EWC establishes a long-term memory preservation mechanism at the parameter level, while GEM maintains optimization consistency between new and historical tasks at the gradient level. These two mechanisms complement each other, ensuring the continuity and discriminability of the feature space during cross-operating-condition and cross-device transfer. By jointly leveraging parameter constraints, gradient constraints, and experience replay, the proposed framework effectively integrates parameter-, gradient-, and data-level compensation, enabling the model to maintain strong generalization capability even in complex, dynamic, and multi-stage industrial scenarios.

## Conclusion

This paper proposes a dual-dimensional constrained continual learning framework with uncertainty quantification for residual life prediction across operating conditions and devices in mechanical systems. Based on a multi-layer Gaussian process architecture, the framework achieves adaptive modeling of complex, non-stationary degradation behavior, ensuring prediction stability and reliability despite varying operating conditions, equipment differences, and sensor layout changes. The framework incorporates parameter importance constraints to safeguard critical structural knowledge, while imposing update direction restrictions through gradient projection strategies. This dual-dimensional collaborative constraint mechanism effectively suppresses catastrophic forgetting during cross-task and cross-device transfer, thereby enhancing the model’s robustness and generalization capability under large distribution shifts. To validate the proposed framework’s efficacy, experimental evaluations were conducted on rolling bearing accelerated degradation datasets and real-world Wind Turbine Gearbox planetary gear operational datasets, encompassing cross-condition and cross-device transfer settings. Comparisons were made against multiple state-of-the-art continual learning approaches. Experimental results demonstrate that the proposed method achieves the lowest AF metrics and optimal RMSE performance across all learning stages, exhibiting outstanding predictive accuracy, knowledge retention capability, and cross-device adaptability throughout the multi-stage continual learning process. Although the proposed framework achieves significant performance improvements in multi-source degradation pattern modeling and cross-device prediction scenarios, it still faces challenges in real industrial settings, such as continuously generated data streams, unpredictable operational condition changes, and long-term distribution drift caused by equipment updates and replacements. Future research may further explore online drift detection, self-identification of task boundaries, and cross-device feature alignment mechanisms. This would enable the model to autonomously adjust its learning strategy and structure without human intervention, thereby enhancing the framework’s scalability, long-term reliability, and practical deployment value in real industrial online operation and maintenance scenarios.

## Data Availability

Data Availability Statement The research data used in this study consist of two components. The XJTU-SY bearing dataset is publicly available at [https://doi.org/10.1177/0954406217710279] (or the original repository link). The wind turbine dataset was generated in our laboratory; due to [institutional policy/confidentiality], this dataset is available from the corresponding author upon reasonable request.
